# ﻿Mountainous millipedes in Vietnam. II. A conspicuous *Tylopus* species from Northern Vietnam (Diplopoda, Polydesmida, Paradoxosomatidae)

**DOI:** 10.3897/zookeys.1091.80094

**Published:** 2022-03-31

**Authors:** Anh D. Nguyen, Katsuyuki Eguchi

**Affiliations:** 1 Institute of Ecology and Biological Resources, Vietnam Academy of Science and Technology, 18, Hoangquocviet Rd., Caugiay District, Hanoi, Vietnam Institute of Ecology and Biological Resources, Vietnam Academy of Science and Technology Hanoi Vietnam; 2 Graduate University of Science and Technology, Vietnam Academy of Science and Technology, 18, Hoangquocviet Rd., Caugiay District, Hanoi, Vietnam Graduate University of Science and Technology Hanoi Vietnam; 3 Department of Biology, Faculty of Science, Tokyo Metropolitan University, Hachioji-shi, Tokyo Prefecture, Japan Tokyo Metropolitan University Hachioji-shi Japan

**Keywords:** Biodiversity, COI, 16S rRNA, mountain fauna, taxonomy

## Abstract

A conspicuous *Tylopus* species is described from Northern Vietnam, namely *T.helicorthomorphoides***sp. nov.** The new species is clearly diagnosed by the gonopodal solenophore completely sheathing the solenomere, both being coiled three times, and the absence of spine z and process h of the gonopod. Fragments of the COI and 16S rRNA genes were extracted, and the phylogenetic analysis also supports the new species.

## ﻿Introduction

The genus *Tylopus* was established by Jeekel (1968) with type species *Agnesiasigma* Attems, 1953. Jeekel (1965, 1968) diagnosed this genus by having well-developed paraterga, first pair of legs without modifications, the presence of tibial and tarsal brushes and adenostyles (= ventral tubercles on the podonomeres), gonopod with somewhat distally enlarged femorite, a distinctly laterally demarcated postfemoral region, postfemorite with 1–3 processes, and both lamina medialis and lamina lateralis well developed. The genus was extensively revised, and morphological terms for the genus *Tylopus* were standardized by Golovatch and Enghoff (1993) and updated by Likhitrakarn et al. (2010, 2016).

*Tylopus* is considered the most species-rich genus within the family Paradoxosomatidae Daday, 1889. Currently, it contains 77 species (Golovatch and Semenyuk 2021; Likhitrakarn et al. 2021; Sierwald and Spelda 2021) distributed from Southern China down to Indonesia and Malaysia, and from Myanmar to Vietnam. They are seemingly dominant in Southeast Asia, especially in Thailand, Laos, and Vietnam, although this may be due to research bias. In Vietnam, 21 species were recorded so far (Attems 1938, 1953; Golovatch 1984; Korsós and Golovatch 1989; Golovatch and Enghoff 1993; Nguyen 2012; Golovatch and Semenyuk 2018, 2021), all of which completely agree with the typical diagnosis for the genus.

As a continuation of our contributions to the millipede fauna of mountainous regions of Vietnam, this work aims to describe a conspicuous *Tylopus* species from northern Vietnam and to discuss relationships between Vietnamese *Tylopus* species.

## ﻿Materials and methods

**Figure 1.**
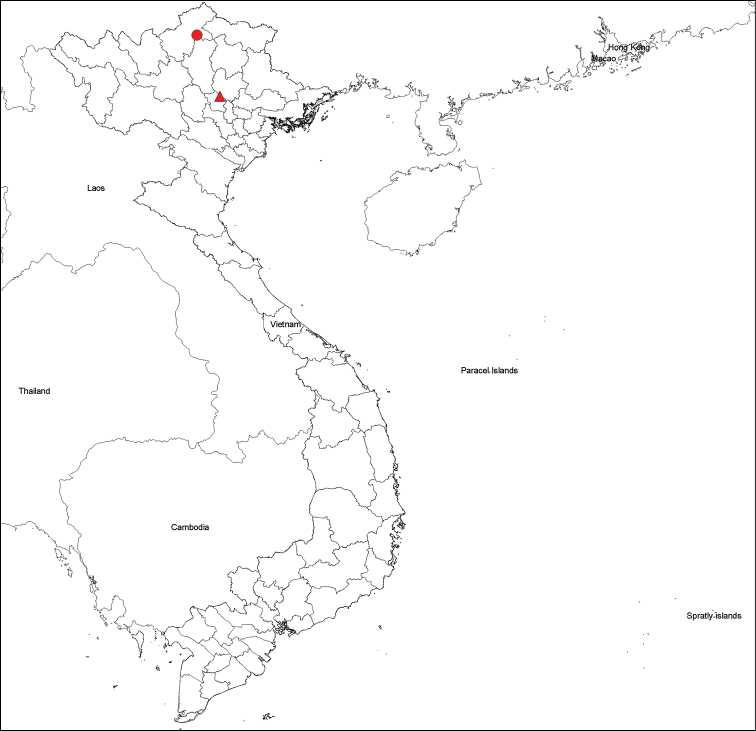
Distribution of *Tylopushelicorthomorphoides* sp. nov. Tam Dao (Red triangular); Bac Me (Red circle)

Material was collected from two localities in Northern Vietnam, Tam Dao National Park and Bac Me Natural Reserve (Fig. 1), and preserved in 90% ethanol. Specimens were observed under an Olympus SZX10 microscope. Images were taken at various focal planes using a Nikon imaging system (Nikon-Br) coupled with a SMZ800N Nikon stereomicroscope. Images were stacked using Helicon Focus version 7.0 and assembled in Adobe Photoshop CS6.

Total DNA was extracted from several midbody legs using the QIAGEN DNeasy Blood & Tissue Kit. Fragments of the mitochondrial cytochrome c oxidase subunit I (COI) and 16S rRNA genes were amplified using two pairs of primers: COI-1F (5’-ACTCTACTAATCATAAGGAT-3’) and COI-1R (5’-TAAACCTCCGGGTGACCAA-3’), 16S-1F (5’-CCGGTTTGAACTCAGATCA-3’) and 16S-1R (5’-TGACTGTTTAGCAAAGACAT-3’). The amplification protocol followed a previously published method by Nguyen et al. (2017). Each successfully amplified and sequenced fragment was assembled using ChromasPro v.2.1.8 and confirmed by BLAST 2.6.0+ searches (Zhang et al. 2000).

All confirmed sequences were aligned using Cluster X ver.2.0 (Larkin et al. 2007), and ambiguous nucleotide sites and gaps were removed using MEGA X (Kumar et al. 2018). The COI sequences were translated into amino acids for confirmation using transversion code in MEGA X. The concatenated dataset (COI+16S) was created from the COI dataset and 16S rRNA dataset using the software GBLOCK 0.91b.

The nucleotide frequencies were statistically calculated using MEGA X. The final dataset contains 956 bp, including 586 bp of the COI gene and 370 bp of the 16S rRNA gene. It has nucleotide frequencies of 26.1, 38.3, 17.5, and 18.0 for A, T, G, and C, respectively. The GC content accounts for 35.5% of total nucleotides. The dataset has 324 parsimony-informative and 403 variable sites.

The phylogenetic tree was reconstructed using the Maximum Likelihood (ML) analysis with the best model resulting the ModelFinder (Kalyaanamoorthy et al. 2017), performed in IQTREE ver.1.6.2 for Windows (Minh et al. 2020). Models with the lowest BIC scores (Bayesian Information Criterion) are considered to describe the best substitution pattern. As a result, the model TIM3+F+G4 was selected (BIC score of 12506.102; -LnL = 6033.252).

All specimens, including holotype and paratypes and DNA vouchers, have been deposited in the Department of Soil Ecology, Institute of Ecology and Biological Resources (**IEBR**), Vietnam Academy of Science and Technology, Hanoi, Vietnam. A total of 24 new sequences were deposited in GenBank, with accession numbers as presented in Table 1.

**Table 1.** Analyzed species, locality data, deposition voucher numbers, and GenBank accession numbers. Accession numbers in bold font are new sequences.

No	Species	Locality	Voucher	16S rRNA	COI
1	*Tylopuscrassipes* Golovatch, 1984	Sapa, Lao Cai	IEBR- Myr 92	**OM978927**	KX096920
2	*Tylopushilaroides* Golovatch, 1984	Cuc Phuong, Ninh Binh	IEBR- Myr 543	**OM978921**	MW384914
3	*Tylopushilaroides* Golovatch, 1984	Cuc Phuong, Ninh Binh	IEBR- Myr 198	KX755588	MW384918
4	*Tylopushilaroides* Golovatch, 1984	Cuc Phuong, Ninh Binh	SVE- Myr 149	**OM978930**	MW384905
5	*Tylopushilaroides* Golovatch, 1984	Cuc Phuong, Ninh Binh	SVE- Myr 173	**OM978931**	MW384904
6	*Tylopushilaroides* Golovatch, 1984	Tam Dao, Vinh Phuc	SVE- Myr 55	**OM978932**	MW384903
7	*Tylopusnodulipes* (Attems, 1953)	Huong Son, Ha Tinh	IEBR- Myr 105	**OM978913**	MW384919
8	*Tylopusnodulipes* (Attems, 1953)	Minh Hoa, Quang Binh	IEBR- Myr 557	**OM978924**	MW384912
9	*Tylopusroseiparaterga* Nguyen, 2012	Ba Vi, Ha Noi	SVE- Myr 70	**OM978933**	MW384902
10	*Tylopussapaensis* Nguyen, 2012	Sa Pa, Lao Cai	IEBR- Myr 93	**OM978928**	MW384908
11	*Tylopusspinisternus* Nguyen, 2012	Bi Doup – Nui Ba, Lam Dong	IEBR- Myr 234	**OM978915**	MW384916
12	*Tylopus* sp.1	Ba Vi, Ha Noi	SVE- Myr 73	**OM978934**	MW384901
13	*Tylopus* sp.1	Ba Vi, Ha Noi	SVE- Myr 74	**OM978935**	MW384900
14	*Tylopus* sp.2	Phong Nha – Ke Bang, Quang Binh	IEBR- Myr 210	**OM978914**	MW384917
15	*Tylopus* sp.2	Phong Nha – Ke Bang, Quang Binh	IEBR- Myr IPE6	**OM978929**	MW384907
16	*Tylopus* sp.3	Sa Pa, Lao Cai	IEBR- Myr 556	**OM978923**	MW384913
17	*Tylopus* sp.4	Son Dong, Bac Giang	IEBR- Myr 509	**OM978919**	MW384915
18	*Tylopushelicorthomorphoides* **sp.nov.**	Tam Dao, Vinh Phuc	IEBR- Myr 603	**OM978925**	MW384910
19	*Tylopus* sp.7	Muong Nhe, Dien Bien	IEBR- Myr 617	**OM978926**	MW384909
20	*Oxidusgigas* (Attems, 1953)	Sapa, Lao Cai	IEBR-Myr 113	KX755581	KX096921
21	*Oxidusgigas* (Attems, 1953)	Duc Xuan, Ha Giang	IEBR-Myr 516	**OM978920**	KX096928
22	*Oxidusriukiaria* (Verhoeff, 1940)	Ryukyu, Japan	IEBR-H500	**OM978918**	KX096926
23	*Oxidusriukiaria* (Verhoeff, 1940)	Ryukyu, Japan	IEBR-H500J	KX755583	KX096927
24	*Oxidusgracilis* (C.L. Koch, 1847)	Taiwan	IEBR- Myr 549	**OM978922**	KX096931
25	*Oxidusgracilis* (C.L. Koch, 1847)	Ryukyu, Japan	IEBR- Myr 466	**OM978916**	KX096924
26	*Oxidusgracilis* (C.L. Koch, 1847)	Ryukyu, Japan	IEBR- Myr 471	**OM978917**	KX096925
27	*Oxidusgracilis* (C.L. Koch, 1847)	USA	IEBR-Myr USA	KX096919	KX096931
28	*Sellanuchezagrandis* (Golovatch, 1984)	Xuan Son, Phu Tho	IEBR-Myr 177	KX755584	KR818296
29	*Sellanuchezahoffmani* Nguyen, 2011	Phong Nha – Ke Bang, Quang Binh	IEBR- Myr 182	KX755585	KR81829
30	*Sellanuchezavariata* (Attems, 1953)	Duc Xuan, Ha Giang	IEBR- Myr 515	KX755586	**OM919709**
	*Antheromorphapumatensis* Nguyen, 2018	Pu Mat, Nghe An	IEBR- Myr IPE3	MG669559	MG669372

## ﻿Taxonomy

### ﻿Order Polydesmida

**Family *Paradoxosomatidae* Daday, 1889**

#### Genus *Tylopus* Attems, 1953

##### ﻿ Tylopus helicorthomorphoides sp. nov.

Taxon classificationAnimaliaPolydesmidaParadoxosomatidae

BED65A57-8088-522D-A2DA-4CBDFDED8643

http://zoobank.org/5CD2F36B-E138-490F-B426-ABFDB47D3A96

**Figure 2.**
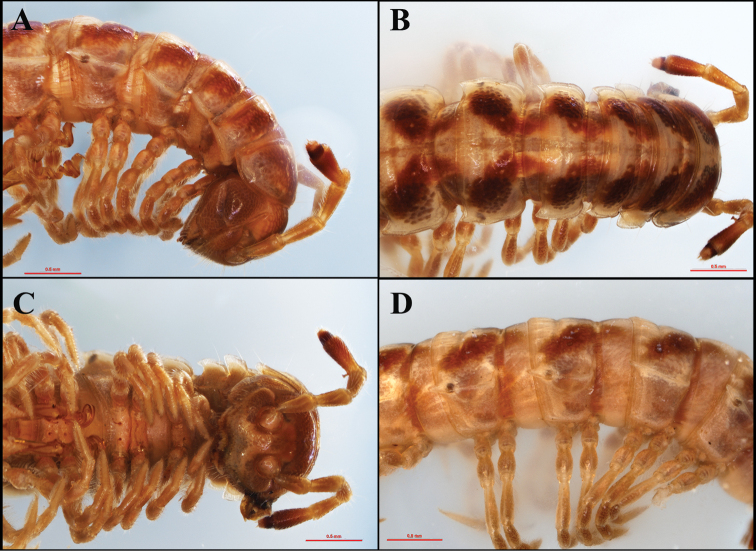
*Tylopushelicorthomorphoides* sp. nov. Holotype (IEBR-Myr 603H). Anterior part of body, lateral view (**A**) dorsal view (**B**) ventral view (**C**) midbody segments, lateral view (**D**).

**Figure 3.**
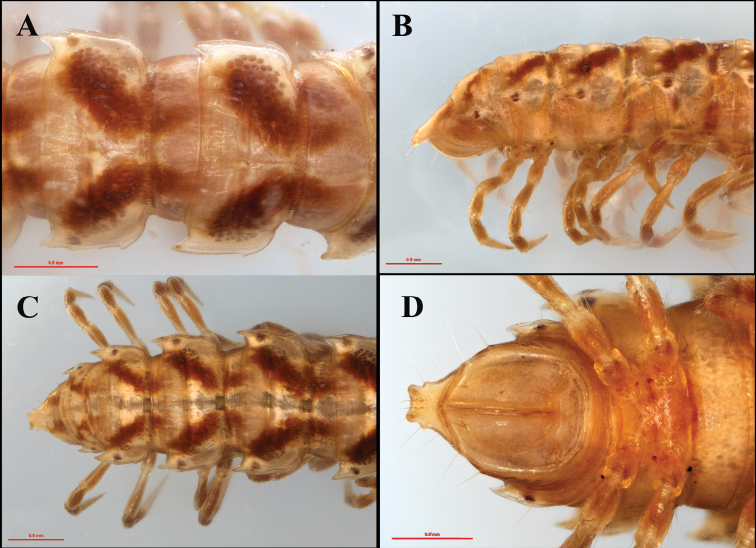
*Tylopushelicorthomorphoides* sp. nov. Holotype (IEBR-Myr 603H). Midbody segments, dorsal view (**A**) posterior part of body, lateral view (**B**) dorsal view (**C**) ventral view (**D**).

**Figure 4.**
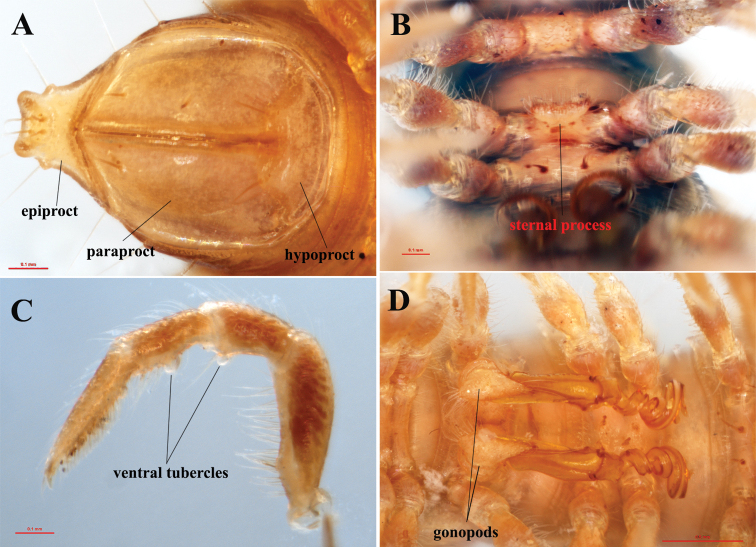
*Tylopushelicorthomorphoides* sp. nov. Holotype (IEBR-Myr 603H). Epiproct, paraproct and hypoproct (**A**) sternal process between coxae 4, ventral view (**B**) leg 6 (**C**) gonopods, ventral view (**D**).

**Figure 5.**
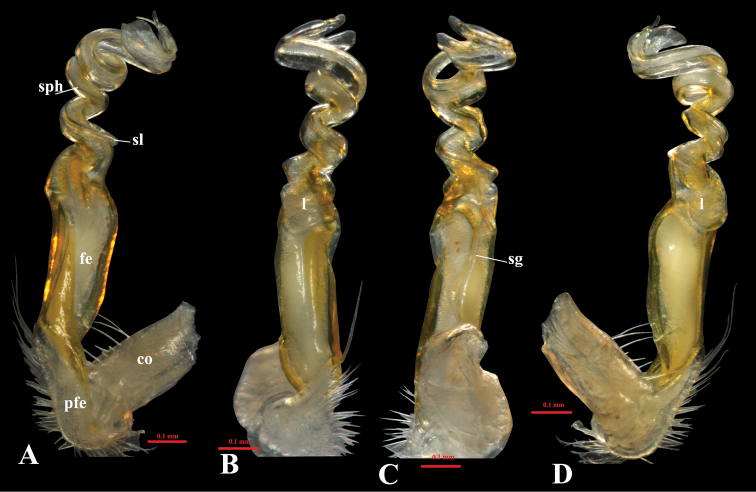
*Tylopushelicorthomorphoides* sp. nov. Holotype (IEBR-Myr 603H). Right gonopod, mesal view (**A**) ventral view (**B**) dorsal view (**C**) lateral view (**D**). Scale bar: 0.1 mm. co = coxite, pfe = prefemorite, fe = femorite, sl = solenomere, sph = solenophore, sg = seminal groove, l = lamella l.

Figs 1 , 2 , 3 , 4 , 5

###### Material examined.

***Holotype*.** Vietnam • 1 male; Vinh Phuc Province, Tam Dao National Park, on the way to Tam Dao 2; 1,100 m a.s.l.; 25 Feb. 2017; Anh D. Nguyen leg.; natural forests; IEBR-Myr 603H.

***Paratypes*.** Vietnam • 2 females; same data as for holotype; IEBR-Myr 603P • 1 male; Vinh Phuc Province, Tam Dao National Park, on way to TV tower; 21.46065°N, 105.64863°E; 1,081 m a.s.l.; 10 Dec. 2019; Hoang Quang Duy leg.; natural forests; IEBR-Myr 876.

***Non-type*.** Vietnam • 1 male; Ha Giang Province, Bac Me Natural Reserve, Minh Ngoc commune, Lung Can village; 22.71814°N, 105.17997°E; 361 m a.s.l.; 12–13 Dec. 2018; Anh D. Nguyen leg.; bushes (IEBR-Myr 809).

###### Diagnosis.

The new species distinctly differs from its congeners in having a three-times spiraled solenophore of the gonopods and postfemoral lamella l present while spine z and process h totally absent. The new species is slightly similar to its congener *T.strongylomoides* (Korsórs & Golovatch, 1989), from the same locality (Tam Dao National Park), in having a twisted solenophore and solenomere. However, the new species obviously differs from *T.strongylosomoides* in having a more strongly coiled solenophore and solenomere (3× vs 1.5×).

Regarding the gonopod conformation, the new species is somewhat similar to species of the genus *Helicorthomorpha* in the twist of both the solenophore and solenomere. However, the postfemoral region of *Helicorthomorpha* members is more elaborate, twisted, and carries no additional processes, whereas that of the new species is spiraled or coiled rather than twisted, and has a postfemoral lamella.

###### Etymology.

The species epithet, *helicorthomorphoides*, is used to emphasize the similarity of the gonopod solenophore between the new species and those of the genus *Helicorthomorpha*.

###### Description.

Body length ~ 14.4 mm (male) and 14.9 mm (female). Width of midbody pro- and metazona ~ 1.2 mm (male), 1.6 mm (female) and 1.6 mm (male), 1.9 mm (female), respectively.

Body generally brownish yellow or darkish yellow except antennomere 7 and metaterga with a darker, median, V-shaped region (or median triangular-shaped area on metaterga) (Figs 2–4).

Head (Fig. 2B, C) slightly smaller than collum, labroclypeous region densely setose. Epicranial suture clearly distinct, dividing frons into two equal parts, with 2+2 setae along suture. Frons convex. Antenna (Fig. 2A–C) short, reaching to approximately tergum 2 laterally. Antennomere 1=7<2=3=4=5<6; antennomere 6 largest, obviously clavate.

Collum (Fig. 2B) oval-shaped, surface smooth, without granulates or tubercles, but with 2+2 setae near anterior margin and 2+2 setae in intermediate area. Paratergum well developed, subtriangular, with broad corner.

Body segments 3<4<2=5–17, thereafter gradually tapering towards telson. Prozonae and metazonae clearly divided by deep, striated waists. Prozonae smooth, shining, yellowish brown, with a median, broad, longitudinal yellow stria (Figs 2B, 3A). Metazonae (Figs 2B, 3A) smooth, shining, with 2+2 setae near anterior margin, two lateral spotted areas, and 2+2 or 3+3 oblong knobs near posterior margin. These knobs more obvious on posterior segments.

Metatergal sulcus starting on segment 4 but clearly present from segment 5, nearly reaching base of paraterga (Fig. 2B). Axial line distinct.

Paraterga (Figs 2A, B, D, 3A–C) well developed, wing-shaped, with at least one setiferous incision near anterior, broadly round corner; caudal corner acute, more pointed from mid-body segment onwards, but never reaching following segment. Caudal corner of paraterga 17–19 very pointed, spine-like (Fig. 3C, D).

Ozopores located inside round hollows at the caudolateral sides of paraterga 5, 7, 9, 10, 12, 13, and 15–19 (Figs 2A, D, 3B).

Pleurites (Figs 2A, D, 3B) smooth, not granulated. Pleurosternal carinae (Fig. 2A) present until segment 8, with a small tubercle-like projection, then gradually reduced or missing on subsequent segments.

***Telson*** (Figs 3C, D, 4A). Epiproct strongly concave, forming two long lateral tubercles. Hypoproct sub-trapeziform, with two separated distolateral setiferous knobs.

***Sterna*.** Cross impression distinct. Sternum 5 with a large rectangular lamina between coxae 4 (Fig. 4B).

Leg short, ~ 1.5× as long as midbody height. Prefemora swollen dorsally. Tarsal brushes present till legs 21, then gradually thinner and absent on subsequent legs. Adenostyles present on postfemora and tibiae, each with a small knob/tubercle (Fig. 4C).

**Figure 6.**
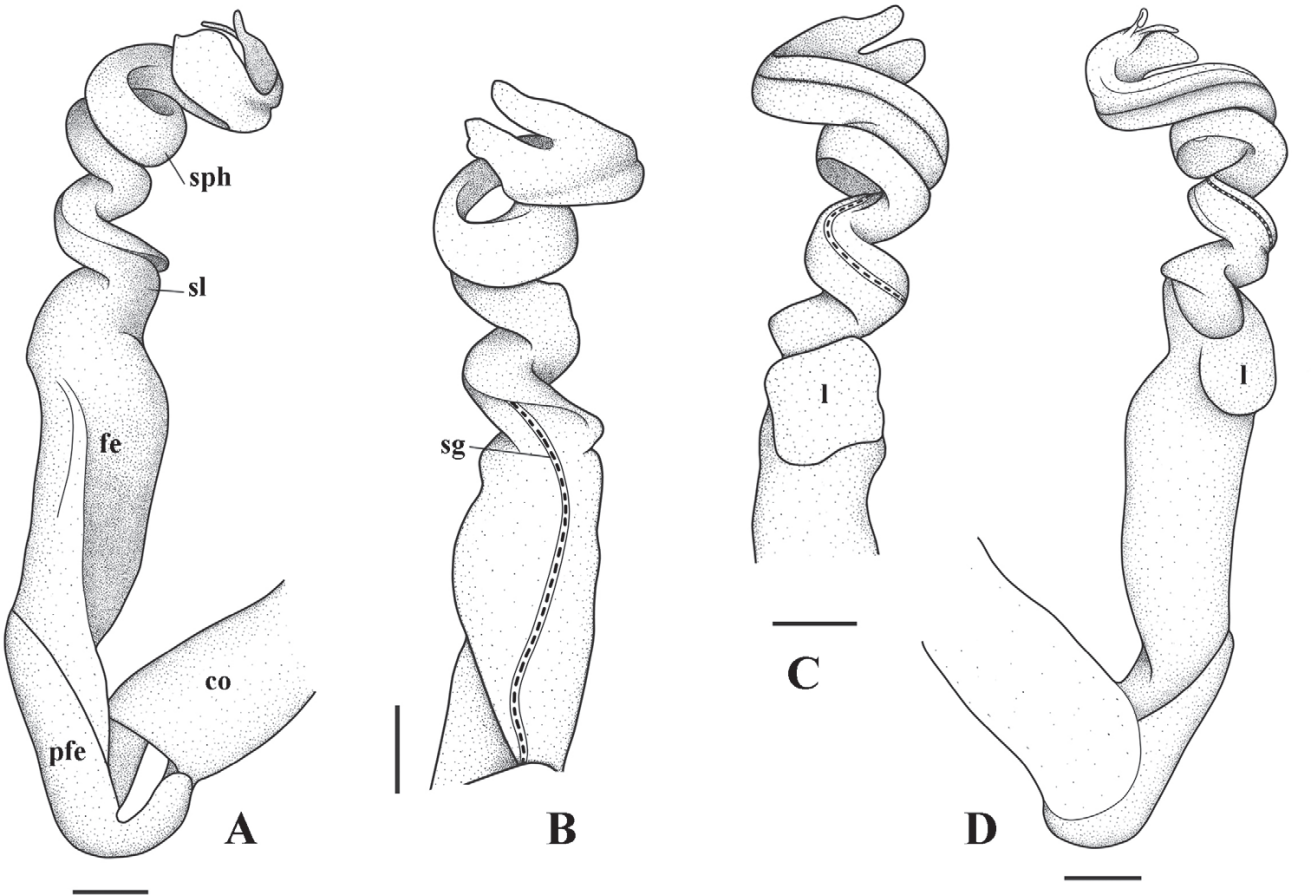
*Tylopushelicorthomorphoides* sp. nov. Holotype (IEBR-Myr 603H). Right gonopod, mesal view (**A**) dorsal view (**B**) ventral view (**C**) lateral view (**D**). Scale bar: 0.1 mm. co = coxite, pfe = prefemorite, fe = femorite, sl = solenomere, sph = solenophore, sg = seminal groove, l = lamella l.

Gonopod suberect, simple (Figs 4D, 5, 6). Coxite short, with sparsely setose distoventral part. Telopodite long and erect. Prefemorite densely setose, separated from femorite by an oblique sulcus laterally. Femorite somewhat enlarged distally and slightly twisted, without modifications or additional processes. Postfemoral region consisting of a solenomere and a solenophore, both completely coiled 3×; lamina l present, spine z and process h missing. Seminal groove running entirely on mesal side of femorite, then entering flagelliform solenomere completely sheathed by solenophore. Tip of gonopod strongly bifid.

###### Genetic distance.

The COI Kimura 2-Parameter (K2P) distance between the new species and other *Tylopus* species was reported in Anh et al. (2021). The distance was from 12.2% to 15.6%. This distance was obviously narrower than the distances between *Sphaerobelum* species (from 20.2% to 24.4%) (Zhao et al. 2020), but more likely similar to the distances between *Glomeris* species (from 11.5% to 17.1%) (Wesener 2015).

###### Phylogenetic analysis.

**Figure 7.**
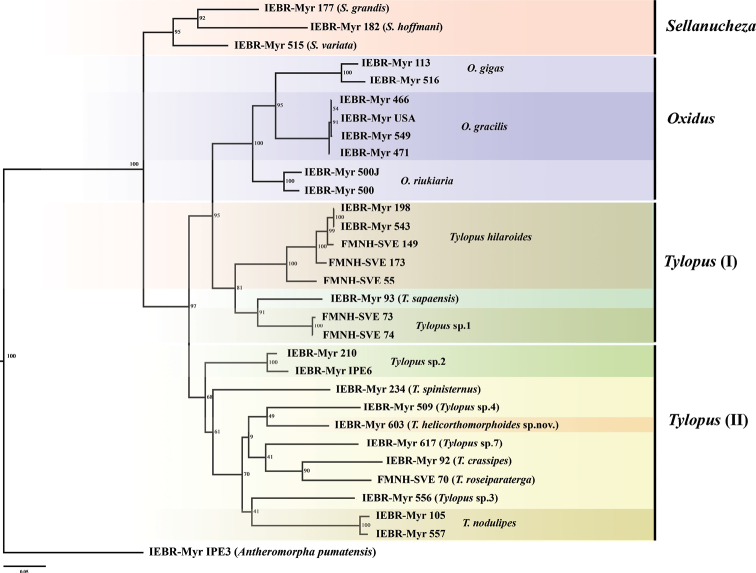
Phylogenetic diagram inferred from the 956 bp COI-16S rRNA dataset using Maximum Likelihood analysis. Numbers shown at nodes are bootstrap values.

The twenty included samples of *Tylopus* members were divided into two different lineages (Fig. 7). The first lineage, *Tylopus* I, consisting of three species, *T.hilaroides*, *T.sapaensis*, and *Tylopus* sp.1, was a sister clade of the genus *Oxidus* with 95% bootstrap support.

The second lineage, *Tylopus* II, was clearly separated from the clade *Oxidus* + *Tylopus* I with high bootstrap support (97%). The new species, *T.helicorthomorphoides* sp. nov., was positioned in this lineage and is closely related to *Tylopus* sp. 4 (IEBR-Myr 509), but with low bootstrap support (49%). In addition, the relationship between *Tylopus* species in the second lineage was very poorly supported (bootstrap less than 70%), except the clade *T.crassipes* + *T.roseiparaterga*, with a bootstrap value of 90%.

## ﻿Discussion

Both Jeekel (1965) and Golovatch and Enghoff (1993) agreed that the gonopod solenophores sheath the flagelliform solenomere for its greater part, curving caudad first, then laterad and finally cephalad in *Tylopus* members. In the new species, the solenophore and solenomere morphology agree with this statement but are completely coiled three times, the gonopod spine z and process h are absent, whereas only postfemoral lamina l is present. Our phylogenetic analysis also supports the taxonomic position of the new species within the genus *Tylopus*.

The genus *Tylopus* is, phylogenetically, divided into two clades as reported above. This also agrees with the results presented by Anh et al. (2021) using only a fragment of the COI gene. It is, therefore, suggested that the genus *Tylopus* needs to be revised accordingly, using both morphological and molecular data. This case may be similar to that of the genus *Desmoxytes* Chamberlin, 1923, which was recently intensively revised by Srisonchai et al. (2018a, 2018b, 2018c, 2018d). Finally, the genus *Hylomus* Cook & Loomis, 1924 has been re-validated, with three new genera proposed for other so-called *Desmoxytes* members (Srisonchai et al. 2018a, 2018b, 2018c, 2018d). Furthermore, the relationship between *Tylopus* species was very poorly supported (less than 70% bootstrap value), especially within the second lineage. More samples and species are required for a better analysis.

The genus *Tylopus* has been found in both lower and higher lands in northern Vietnam, but it has only been recorded in higher lands in southern Vietnam, and has never been found in the Mekong delta (Nguyen 2012; Golovatch and Semenyuk 2018, 2021). The distributional pattern of this genus in Vietnam is similar to that in Thailand, where *Tylopus* species are mostly found in mountainous regions with altitudes of more than 500 m in northern and central Thailand (Likhitrakarn et al. 2010). The highest recorded altitude is 2,300m in Ngoc Linh Mt. (Vietnam) for *Tylopushilaris* (Attems, 1937) and *T.phanluongi* Nguyen, 2012. Furthermore, as mentioned by Nguyen et al. (2019, 2021), the mountainous region of Vietnam harbors a rich biodiversity in Vietnam, but it is far from completely known because access to the region is difficult and intensive surveys are still lacking.

## ﻿Conclusion

With the new species described herein, the number of *Tylopus* species known for Vietnam increases to 22. However, this number is still far from representing the true diversity of the genus in Vietnam. More intensive surveys will reveal more new discoveries, especially in the diverse high mountainous regions of Vietnam, which remain underexplored.

## Supplementary Material

XML Treatment for Tylopus helicorthomorphoides

## Supplementary materials

Supplementary material 1 The COI-16S rRNA dataset Data type DNA data Explanation note The COI-16S rRNA dataset contains 956 bp, including 586 bp of the COI gene and 370 bp of the 16S rRNA gene. https://binary.pensoft.net/file/665850This dataset is made available under the Open Database License (http://opendatacommons.org/licenses/odbl/1.0/). The Open Database License (ODbL) is a license agreement intended to allow users to freely share, modify, and use this Dataset while maintaining this same freedom for others, provided that the original source and author(s) are credited.Anh D. Nguyen, Katsuyuki Eguchi
